# Clinical and laboratory evaluation in horses submitted to intracecal fluid therapy administered in two different rates

**DOI:** 10.3389/fvets.2025.1637033

**Published:** 2026-01-12

**Authors:** Brenda Ventura Lopes Carvalho, Maria Carolina Neves de Souza, Nadyne Souza Moreira, Julia Parisi Marliere, João Victor Mesquita Mota, Lucas Drumond Bento, Anaïs de Castro Benitez, Marcel Ferreira Bastos Avanza, Raffaella Bertoni Cavalcanti Teixeira, José Ricardo Barboza Silva, Rinaldo Batista Viana, Bruno Moura Monteiro, Pedro Paulo Maia Teixeira, Ramiro Toribio, Hélio Cordeiro Manso Filho, José Dantas Ribeiro Filho

**Affiliations:** 1 Department of Veterinary, Federal University of Viçosa, Viçosa, Brazil; 2 Institute of Animal Health and Production, Federal Rural University of the Amazon, Belém, Brazil; 3 Institute of Veterinary Medicine, Federal University of Pará, Castanhal, Brazil; 4 Department of Veterinary Clinical Sciences, The Ohio State University, Columbus, OH, United States; 5 Department of Animal Science, Federal Rural University of Pernambuco, Recife, Brazil

**Keywords:** blood gas analysis, cecum cannulation, equine, rehydration, serum biochemistry

## Abstract

**Introduction:**

Two routes of administration for electrolyte solutions are commonly used in horses with fluid, electrolyte, and acid–base imbalances: intravenous and nasogastric. Despite the frequent use of these routes, there are situations in which they cannot be applied. In such cases, intracecal fluid therapy represents a viable alternative, as this route enables the administration of enteral electrolyte solutions even in animals lacking normal anterior gastrointestinal function. This study aimed to evaluate the effects of a neutral hypotonic enteral electrolyte solution administered intracecally at two different infusion rates in horses experimentally subjected to a 24-h fasting period.

**Methods:**

This study employed a crossover design in which six experimentally dehydrated horses underwent two treatments: Treat10 (10 mL kg^−1^ h^−1^) and Treat15 (15 mL kg^−1^ h^−1^) with a single neutral solution. Horses were subjected to cecal cannulation via video laparoscopy using a modified Hasson technique to insert a Foley catheter for solution administration. Samples were collected at T-24 h (baseline, at the start of the water and food deprivation phase), T0h (at the end of the deprivation phase and the beginning of the fluid therapy), T4h (4 h after the start of fluid therapy), T8h (8 h after the start of fluid therapy), T12h (twelve hours after the start of fluid therapy), and T24h (twelve hours after the end of fluid therapy). Blood gas analysis and measurements of serum osmolarity, sodium, potassium, chloride, calcium, magnesium, phosphorus, fibrinogen, urea, creatinine, total protein, lactate, and glucose concentrations were performed. Urine samples were analyzed for concentrations of urea, creatinine, sodium, potassium, chloride, calcium, magnesium, and phosphorus.

**Results:**

During the fluid therapy phase in animals from both groups, a progressive decrease in serum urea and potassium concentrations was recorded. A decrease in urinary specific gravity, urea and creatinine was also detected during the same period.

**Conclusion:**

Intracecal administration of electrolyte solutions at rates of 10 mL (Treat10) and 15 mL (Treat15) over 12 h was effective and safe. The electrolyte solution used did not cause significant alterations in electrolyte or acid–base balance, suggesting that its composition is appropriate.

## 1 Introduction

Modern practices in horse breeding, work, and sports increasingly diverge from the animals’ natural behavior, often contributing to the development or predisposition to various diseases. Many of these pathological conditions are associated with inflammatory processes. In horses, this may lead to reduced water and feed intake. A decrease in intake, combined with inflammation, often results in varying degrees of dehydration, especially when the gastrointestinal tract is involved, as seen in conditions such as colic syndrome, enteritis, and diarrhea (1).

Dehydration is often accompanied by disturbances in electrolyte and acid–base balance. Therefore, formulas used to correct dehydration must address not only volume replacement. These solutions must contain adequate amounts of electrolytes to restore balance without causing a secondary acid–base disturbance (2). In horses, two administration routes are commonly used: intravenous (IV) and nasogastric (NG). The IV route allows for rapid volume replacement and is primarily indicated in cases of severe dehydration, while the NG route is recommended for mild to moderate cases, as it requires absorption to promote volume expansion. The NG route is frequently preferred due to economic considerations, as it represents the least expensive option (1, 3).

Despite the routine use of these routes, there are situations in which they cannot be employed. When horses have gastric reflux, the nasogastric route should be avoided, making intravenous administration the only option. However, prolonged venous cannulation can lead to phlebitis, and IV treatment can also be an expensive choice (3). In such cases, the intracecal route in horses represents an alternative to the intravenous and nasogastric routes that deserves further consideration and study, as it allows the use of enteral electrolyte solutions even in animals without preserved anterior gastrointestinal function. Furthermore, the novelty of this research must be highlighted. The therapy with enteral electrolyte solution administered intracecally at different rates and continuous flow has not been previously described.

Prior to the present study, this research group has tested different infusion rates of an enteral electrolyte solution administered intracecally in adult horses by pilot studies seeking to determine effective and safe rates for rehydrating this class of animals. Thus, this study aimed to evaluate the effects of a neutral hypotonic enteral electrolyte solution administered intracecally at two different infusion rates in horses experimentally subjected to a 24-h fasting period. The hypothesis is that an infusion rate of 10 mL kg^−1^ h^−1^ is safer and equally effective compared to the 15 mL kg^−1^ h^−1^ rate, both administered for 12 h in continuous flow.

## 2 Materials and methods

### 2.1 Ethical approval and animals

This study was conducted under the approval of the Animal Ethics and Welfare Committee of the Federal University of Viçosa (protocol no. 17/2015) and was conducted between March and June 2022. Six healthy horses between 5 and 15 years and weighing between 295 and 445 kg, were used. The animals underwent cecal cannulation via video laparoscopy, using an adaption of the Hasson technique. This procedure involved the placement of a Foley catheter into the cecum, which was subsequently used for solution administration (4). Prior to the Foley catheter placement procedure, the animals underwent an adaptation period. All animals were housed in individual stalls and fed fresh *Pennisetum purpureum*, *Cynodon* spp., commercial concentrate, mineral salt, and water *ad libitum*. It should be noticed that intracecal fluid therapy was performed immediately after placing the cannula in the cecum, and this cannula was inserted into the animal’s cecum only once, at the beginning of the trial remaining there until the end, and was used twice, once for each treatment phase. There was no surgical recovery period.

### 2.2 Experimental design

This study consisted of a 6 × 2 crossover design, where six horses were subjected to two enteral fluid therapy (EFT) treatments, administered seven days apart. Animals’ allocation to the treatment sequence was randomized. Both treatments consisted of the same enteral electrolyte solution (EES), administered at two different infusion rates: Treat10 (10 mL kg^−1^ h^−1^) and Treat15 (15 mL kg^−1^ h^−1^), each administered over a 12-h period.

Samples were collected at T-24 h (baseline, at the start of the water and food deprivation phase), T0h (at the end of the deprivation phase and the beginning of the fluid therapy), T4h (4 h after the start of fluid therapy), T8h (8 h after the start of fluid therapy), T12h (twelve hours after the start of fluid therapy), and T24h (twelve hours after the end of fluid therapy).

All animals were deprived of food and water for 24 h before treatment (T-24 h to T0h) and throughout the fluid therapy phase (T0h to T12h). At the end of the fluid therapy phase, free-choice hay and water were offered (T12h to T24h).

The composition of the enteral electrolyte solution used in the experiment consisted of 4.5 g of sodium chloride, 0.5 g of potassium chloride, 1 g of calcium acetate, 0.2 g of magnesium chloride, and 5 g of dextrose diluted in 1,000 mL of water. The measured osmolarity of the enteral electrolyte solution was 238 mOsm L^−1^.

All animals received the solution in a continuous flow rate through the intracecal catheter (18 Fr Foley silicon catheter Well Lead Medical Co.), connected to a 20-liter reservoir and a 5-meter-long infusion set equipped with a drip chamber and a flow regulator. At the end of the fluid therapy period, the EFT system was detached, and the Foley catheter was sealed to prevent leakage of cecal contents and the entry of air into the cecum.

### 2.3 Clinical evaluations e laboratory analysis

Cardiac rate was obtained in beats per minute (bpm) through auscultation with stethoscope for one minute. The same technique was used to obtain the respiratory rate, in respiratory movements per minute (rmpm). Intestinal auscultation was performed by dividing the abdomen into four quadrants, ventral and dorsal of left and right sides. After one minute of auscultation, the motility of each quadrant was rated on a scale from one to three. The overall intestinal motility consisted of the summatory of all quadrants (resulting in a scale of four to twelve). A small area of the 17th intercostal space.

of the animals was trichotomized, bilaterally and equivalently, which served as a reference point for passing a measuring tape to evaluate the abdominal circumference (in centimeters).

Blood samples were collected in K2 EDTA tube to measure packed cell volume, by a hematological analyzer (Hematoclin 2.8 Vet - BioClin©). Blood samples were collected via jugular venipuncture and placed into sodium fluoride tubes for plasma biochemistry, and clot activator tubes for serum biochemistry. Blood samples were collected into heparinized syringes for venous blood gas analysis.

Serum osmolarity was determined using an Osmometer Model 3,320 (Advanced Instruments Inc.). Biochemical analyses were performed using the BioClin 2,200 (BioClin Quibasa Ltda.) and the HumaStar 300 SR (Human©) analyzers. Variables measured included sodium, potassium, chloride, calcium, magnesium, phosphorus, fibrinogen, urea, creatinine, and total protein. Plasma concentrations of L-lactate and glucose were also determined. Acid–base balance and blood gas analysis were assessed using the ABL80 Flex analyzer (Radiometer Medical ApS©), with measurements of blood pH, pCO_2_, HCO_3_^−^, and base excess (BE). Anion gap (AG) and strong ion difference (SID) were calculated using the following formulas: AG = (Na^+^ + K^+^) – (Cl^−^ + HCO_3_^−^); SID = (Na^+^ + K^+^) – (Cl^−^).

Urine samples were collected from spontaneous urination and submitted for analysis. Urinary pH was measured using the DLA-PH analyzer (Del Lab®), and urinary specific gravity was determined with a refractometer (ATC model RTP-12). Urine samples were analyzed for urea, creatinine, sodium, potassium, chloride, calcium, magnesium, and phosphorus levels.

### 2.4 Statistical analysis

Descriptive statistical analysis was expressed as mean ± SD for continuous variables and percentage (%) for binomial variables. These data were processed using SAS software (SAS/STAT, SAS Institute Inc., Cary, NC, USA, version 9.3).

Inferential statistical analysis was performed using repeated measures ANOVA to compare the means and standard deviations of the animals (*n* = 6) across treatments (*n* = 2), time points (*n* = 6), and replicates (*n* = 2) (each animal undergoes one treatment in the first replicate and the other treatment in the second replicate, in a Cross-Over design). The continuous response variables were subjected to the response scaling test through the Guided Data Analysis solution of SAS 9.3. Variables that did not follow these assumptions were transformed accordingly.

The statistical model included the independent variables (treatment, time, treatment*time interaction, and repetition), a random (animal nested within time), and dependent variables (clinical and laboratory parameters). Group comparisons were conducted using the Tukey test via the Least Square Means (LSMeans) procedure in SAS. A significance level of 5% (*p* < 0.05) was adopted for all statistical tests. Graphs were generated using SigmaPlot software (Systat Software GmbH, Erkrath, Germany, version 12.0).

## 3 Results

Packed cell volume and total protein did not show a significant difference during the fluid therapy phase in animals from both treatments (*p* > 0.05, Table 1). A slight but significant increase in abdominal circumference values was observed in animals that received Treat15.

**Table 1:** Mean values ± standard deviation for heart rate (HR), respiratory rate (RR), packed cell volume (PCV), total serum protein (TSP), intestinal motility, and abdominal circumference in horses submitted to enteral fluid therapy in continuous flow via intracecal route.

Variable	Treatment	Time					
		T-24 h	T0h	T4h	T8h	T12h	T24h
HR bpm	Treat10	32.33 ± 4.63^bc^	30.33 ± 4.63^c^	33.00 ± 3.74b^c^	35.67 ± 3.88^bac^	34.67 ± 3.27^bac^	34.00 ± 3.58^bac^
	Treat15	33.00 ± 4.69^bc^	31.67 ± 5.13^bc^	31.00 ± 5.13b^c^	36.33 ± 6.98^bac^	39.67 ± 7.74^a^	37.00 ± 7.56^ba^
RR rpm	Treat10	10.33 ± 3.20^a^	9.33 ± 2.07^a^	8.67 ± 1.03^a^	10.67 ± 2.07^a^	9.33 ± 2.07^a^	12.67 ± 3.72^a^
	Treat15	11.83 ± 4.40^a^	10.33 ± 1.97^a^	9.00 ± 2.45^a^	9.67 ± 2.94^a^	12.00 ± 1.26^a^	12.67 ± 4.13^a^
PCV %	Treat10	27.63 ± 3.15^a^	27.95 ± 3.94^a^	24.6 ± 2.83^a^	24.58 ± 2.33^a^	24.28 ± 2.97^a^	25.62 ± 3.68^a^
	Treat15	27.17 ± 1.83^a^	27.83 ± 1.83^a^	25.2 ± 3.52^a^	24.44 ± 3.80^a^	24.50 ± 4.18^a^	26.67 ± 1.86^a^
TSP g dL^−1^	Treat10	6.77 ± 0.52^a^	7.08 ± 0.47^a^	6.62 ± 0.40^a^	6.50 ± 0.38^a^	6.47 ± 0.40^a^	6.82 ± 0.37^a^
	Treat15	6.93 ± 0.42^a^	7.17 ± 0.42^a^	6.65 ± 0.38^a^	6.53 ± 0.29^a^	6.45 ± 0.50^a^	7.00 ± 0.21^a^
Intestinal motility	Treat10	7.33 ± 1.63^ba^	5.83 ± 1.83^ba^	6.33 ± 1.97^ba^	5.50 ± 1.22^ba^	5.33 ± 2.42^b^	6.33 ± 1.86^ba^
	Treat15	7.33 ± 1.63^ba^	5.33 ± 1.21b^a^	6.50 ± 1.97^ba^	6.50 ± 1.97^ba^	6.50 ± 2.07^ba^	7.50 ± 0.84^a^
Abdominal circumference cm	Treat10	169.50 ± 5.50^dec^	166.17 ± 5.60^e^	172.33 ± 5.85^bdec^	174.67 ± 7.12^bdac^	176.00 ± 6.32^bac^	172.00 ± 6.07^bdec^
	Treat15	169.67 ± 6.89^dec^	168.33 ± 4.97^de^	173.67 ± 5.85^bdac^	177.33 ± 5.32^ba^	179.50 ± 4.89^a^	170.67 ± 5.01^bdec^

Values represented by different letters indicate statistical difference (*p* < 0.05).

Serum urea levels changed significantly over time (*p* < 0.0001), increasing at T0 and progressively decreasing during fluid therapy. Treatments did not affect urea concentrations. Creatinine showed no significant variation over time. Glucose exhibited significant time-related variation (*p* < 0.0001), with the highest plasma glucose values observed at T24 in animals from both treatments (*p* < 0.0001). Lactate concentrations were consistently higher (*p* = 0.0002) in Treat15 compared to Treat10 but did not change over time. The electrolytes sodium (Na^+^), chloride (Cl^−^), ionized calcium (iCa^++^), and magnesium (Mg^++^) showed no significant differences in any comparison. Potassium (K^+^) significantly changed over time (*p* < 0.0001), with a noticeable decrease during fluid therapy. Phosphorus (P) also varied over time (*p* < 0.0001), with a decrease after the end of fluid therapy, at T24 (Table 2).

**Table 2:** Mean values ± standard deviation for serum and plasma biochemistry: Urea, creatinine, glucose, lactate, ionized calcium (iCa^++^), phosphorus (P), magnesium (Mg^++^), chloride (Cl^−^), sodium (Na^+^), potassium (K^+^) and serum osmolarity in horses submitted to enteral fluid therapy in continuous flow via intracecal route.

Variable	Treatment	Time					
		T-24H	T0H	T4H	T8H	T12H	T24H
Urea mg dL^−1^	Treat10	23.83 ± 6.11^dc^	32.17 ± 4.12^a^	30.50 ± 5.61^ba^	25.00 ± 6.10^bc^	20.17 ± 5.12^dc^	23.50 ± 6.86^dc^
	Treat15	24.17 ± 3.31^dc^	33.00 ± 5.14^a^	30.50 ± 5.96^ba^	24.50 ± 4.93^c^	18.33 ± 4.55^d^	20.33 ± 3.56^dc^
Creatinine mg dL^−1^	Treat10	1.04 ± 0.31^a^	1.05 ± 0.27^a^	1.01 ± 0.31^a^	0.99 ± 0.33^a^	0.98 ± 0.31^a^	0.99 ± 0.33^a^
	Treat15	0.97 ± 0.12^a^	1.06 ± 0.18^a^	0.99 ± 0.20^a^	0.95 ± 0.18^a^	0.93 ± 0.17^a^	0.94 ± 0.14^a^
Glucose mg dL^−1^	Treat10	91.00 ± 4.82^bc^	88.67 ± 4.37^bcd^	78.83 ± 9.83^d^	83.33 ± 3.39^cd^	83.33 ± 4.63^cd^	98.50 ± 7.26^ba^
	Treat15	89.50 ± 3.78^bcd^	88.33 ± 2.25^bcd^	83.83 ± 5.85^cd^	86.00 ± 4.43^cd^	89.83 ± 5.56^bc^	105.50 ± 26.67^a^
Lactate mg dL^−1^	Treat10	8.83 ± 3.31^bdc^	7.50 ± 1.05^bdc^	7.50 ± 1.87^bdc^	7.83 ± 1.72^bdc^	7.00 ± 1.26^d^	7.17 ± 1.72^dc^
	Treat15	11.00 ± 1.79^a^	8.50 ± 1.05^bdc^	9.17 ± 2.14^bac^	9.00 ± 1.67^bdac^	9.50 ± 2.43^ba^	8.83 ± 0.75^bdc^
iCa^++^ mmol L^−1^	Treat10	1.64 ± 0.09^a^	1.57 ± 0.11^a^	1.58 ± 0.06^a^	1.50 ± 0.06^a^	1.53 ± 0.11^a^	1.61 ± 0.03^a^
	Treat15	1.61 ± 0.07^a^	1.61 ± 0.07^a^	1.53 ± 0.07^a^	1.53 ± 0.10^a^	1.48 ± 0.10^a^	1.56 ± 0.13^a^
P mg dL^−1^	Treat10	2.60 ± 0.51^a^	2.78 ± 0.63^a^	2.93 ± 0.66^a^	2.88 ± 0.58^a^	2.63 ± 0.43^a^	1.90 ± 0.46^b^
	Treat15	2.87 ± 0.31^a^	2.70 ± 0.29^a^	2.75 ± 0.34^a^	2.78 ± 0.25^a^	2.92 ± 0.58^a^	1.88 ± 0.51^b^
Mg^++^ mg dL^−1^	Treat10	1.67 ± 0.27^ba^	1.65 ± 0.19^ba^	1.57 ± 0.08^ba^	1.30 ± 0.11^b^	1.22 ± 0.10b^a^	1.52 ± 0.15^ba^
	Treat15	1.65 ± 0.31^ba^	1.67 ± 0.23^ba^	1.58 ± 0.25^a^	1.37 ± 0.19^ba^	1.20 ± 0.17b^a^	1.57 ± 0.19^ba^
Cl^−^ mmol L^−1^	Treat10	97.83 ± 2.14^a^	99.17 ± 2.93^a^	98.17 ± 1.83^a^	98.67 ± 2.88^a^	99.17 ± 2.64^a^	99.17 ± 2.79^a^
	Treat15	97.83 ± 2.04^a^	99.17 ± 2.86^a^	98.50 ± 2.59^a^	98.83 ± 1.94^a^	99.50 ± 1.52^a^	100.50 ± 4.14^a^
Na^+^ mmol L^−1^	Treat10	139.00 ± 1.67^ba^	140.33 ± 1.86^a^	140.50 ± 1.87^a^	140.17 ± 1.94^a^	139.67 ± 1.97^ba^	137.17 ± 2.40^b^
	Treat15	138.50 ± 1.52^ba^	138.50 ± 1.97^ba^	140.00 ± 2.00^a^	140.67 ± 0.82^a^	141.33 ± 0.82^a^	138.5 ± 2.59^ba^
K^+^ mmol L^−1^	Treat10	3.94 ± 0.33^bac^	3.88 ± 0.17^bdac^	3.54 ± 0.23^e^	3.53 ± 0.21^e^	3.70 ± 0.25^dec^	3.78 ± 0.40^bdec^
	Treat15	4.07 ± 0.13^ba^	4.12 ± 0.34^a^	3.63 ± 0.12^dec^	3.59 ± 0.15^de^	3.50 ± 0.37^e^	3.62 ± 0.44^dec^
Osmolarity mOsm L^−1^	Treat10	289.67 ± 7.74^ba^	287.83 ± 4.83^bdac^	289.17 ± 4.02^bac^	288.00 ± 3.22^bdac^	282.33 ± 7.34^d^	283.17 ± 5.38^dc^
	Treart15	288.00 ± 6.00^bdac^	292.33 ± 4.27^a^	288.33 ± 2.42^bdac^	288.00 ± 3.85^bdac^	287.5 ± 9.77^bdac^	286.00 ± 5.87^bdc^

Values represented by different letters indicate statistical difference (*p* < 0.05).

Serum osmolarity did not differ significantly over time or between treatments (Table 2). Venous blood pH and pCO₂ also remained stable. Plasma HCO₃^−^ and base excess (BE) varied significantly over time (*p* = 0.0059 and *p* = 0.0001, respectively), with decreasing trends starting at T8 and continuing post-treatment. AG and SID showed no significant changes over time or between treatments (Table 3).

**Table 3:** Mean values ± standard deviation for pH, carbon dioxide pressure (pCO_2_), bicarbonate concentration (HCO_3_^−^), base excess (BE), anion gap (AG), and strong ion difference (SID) in horses submitted to enteral fluid therapy in continuous flow via intracecal route.

Variable	Treatment	Time					
		T-24H	T0H	T4H	T8H	T12H	T24H
pH	Treat10	7.44 ± 0.02^a^	7.42 ± 0.03^a^	7.42 ± 0.02^a^	7.42 ± 0.02^a^	7.42 ± 0.01^a^	7.39 ± 0.02^a^
	Treat15	7.41 ± 0.02^a^	7.39 ± 0.03^a^	7.42 ± 0.02^a^	7.42 ± 0.02^a^	7.41 ± 0.02^a^	7.41 ± 0.01^a^
pCO_2_ mm Hg	Treat10	41.22 ± 3.61^ba^	40.90 ± 5.44^ba^	43.05 ± 1.88^ba^	42.53 ± 3.08^ba^	41.98 ± 2.80^ba^	40.17 ± 3.44^b^
	Treat15	43.77 ± 4.61^ba^	42.67 ± 1.72^ba^	42.32 ± 2.17^ba^	43.70 ± 3.62^ba^	44.15 ± 2.87^a^	40.33 ± 3.65^ba^
HCO_3_^−^ mmol L^−1^	Treat10	27.23 ± 3.03^a^	27.83 ± 4.62^a^	27.40 ± 2.15^a^	26.88 ± 1.89^ba^	26.08 ± 1.56^bac^	23.58 ± 1.06^c^
	Treat15	26.8 ± 2.19^ba^	26.12 ± 1.35^bac^	27.30 ± 1.33^a^	27.80 ± 1.60^a^	27.22 ± 1.47^a^	24.58 ± 2.31^c^
BE mmol L^−1^	Treat10	3.20 ± 2.77^a^	2.07 ± 1.22^ba^	3.10 ± 2.13^a^	2.85 ± 1.68^a^	2.03 ± 1.39^ba^	−0.47 ± 0.63^c^
	Treat15	2.45 ± 1.89^ba^	1.77 ± 1.29^ba^	3.08 ± 1.31^a^	3.62 ± 1.15^a^	2.98 ± 1.14^a^	0.60 ± 1.98^bc^
AG mmol L^−1^	Treat10	17.87 ± 1.02^a^	18.88 ± 0.97^a^	18.47 ± 0.86^a^	18.15 ± 1.03^a^	18.11 ± 0.76^a^	18.20 ± 1.24^a^
	Treat15	17.94 ± 2.25^a^	17.34 ± 3.79^a^	17.83 ± 2.3^a^	17.62 ± 1.70^a^	18.12 ± 2.22^a^	17.03 ± 2.62^a^
SID mmol L^−1^	Treat10	45.10 ± 2.88^a^	45.05 ± 2.16^a^	45.87 ± 1.82^a^	45.03 ± 2.24^a^	44.20 ± 1.52^a^	41.78 ± 1.06^a^
	Treat15	44.74 ± 1.68^a^	43.46 ± 3.2^a^	45.13 ± 2.6^a^	45.42 ± 2.31^a^	45.33 ± 1.79^a^	41.62 ± 2.93^a^

Values represented by different letters indicate statistical difference (*p* < 0.05).

Urine specific gravity showed a decreasing trend during fluid therapy, but this change was not statistically significant. In contrast, urinary pH showed significant variation (*p* = 0.0008), with a decrease after fasting, an increase during fluid therapy, and a subsequent drop between T12 and T24 (Table 4).

**Table 4:** Mean values ± standard deviation for urinary analysis: specific gravity, pH, urea, creatinine, calcium (UrCa^++^), phosphorus (UrP), magnesium (UrMg^++^), chloride (UrCl^−^), sodium (UrNa^+^), and potassium (UrK^+^) in horses submitted to enteral fluid therapy in continuous flow via intracecal route.

Variable	Treatment	Time					
		T-24H	T0H	T4H	T8H	T12H	T24H
Specific gravity	Treat10	1021.00 ± 15.10^ba^	1026.80 ± 16.65^a^	1025.50 ± 4.12^a^	1012.00 ± 10.04^ba^	1006.33 ± 3.20^b^	1025.00 ± 7.07^ba^
	Treat15	1017.25 ± 7.63^ba^	1027.60 ± 13.45^a^	1018.80 ± 13.75^ba^	1005.67 ± 2.94^b^	1004.67 ± 0.82^b^	1015.2 ± 6.87^ba^
pH	Treat10	8.46 ± 0.34^a^	7.41 ± 1.02^bc^	8.17 ± 0.75^ba^	8.15 ± 0.46^ba^	8.07 ± 0.37^ba^	6.82 ± 1.25^c^
	Treat15	7.89 ± 0.56^ba^	7.54 ± 0.78^bc^	8.09 ± 0.58^ba^	7.90 ± 0.20^ba^	8.03 ± 0.23^ba^	7.04 ± 0.55^c^
UrUrea mg dL^−1^	Treat10	1402.97 ± 1550.94^ba^	1654.34 ± 1166.55^bc^	1980.38 ± 1315.26^a^	644.57 ± 663.53^bc^	414.33 ± 317.08^c^	1438.05 ± 673.66^bac^
	Treat15	1135.19 ± 307.49^bac^	2696.39 ± 1452.33^a^	1757.33 ± 1380.08^a^	337.66 ± 149.84^bc^	244.39 ± 83.53^c^	823.18 ± 435.82^bc^
UrCreatinine mg dL^−1^	Treat10	215.37 ± 224.37^ba^	235.54 ± 136.7^ba^	107.61 ± 69.97^bac^	48.23 ± 65.06^bc^	35.70 ± 30.82^c^	151.77 ± 76.04^bac^
	Treat15	84.99 ± 28.19^bac^	340.07 ± 239.34^a^	149.74 ± 135.75^bac^	20.54 ± 7.65^bc^	14.26 ± 1.97^c^	64.18 ± 35.56^bc^
UrCa^++^ mg dL^−1^	Treat10	25.20 ± 25.55^a^	10.86 ± 8.06^a^	4.34 ± 4.93^a^	3.27 ± 1.14^a^	4.68 ± 2.05^a^	33.57 ± 20.65^a^
	Treat15	20.60 ± 29.99^a^	14.30 ± 20.75^a^	5.75 ± 6.80^a^	3.33 ± 1.82^a^	3.97 ± 0.78^a^	48.95 ± 49.52^a^
UrP mg dL^−1^	Treat10	3.20 ± 0.61^bdc^	7.40 ± 3.89^a^	5.21 ± 1.43^bac^	4.06 ± 1.52^bdc^	3.68 ± 0.45^dc^	4.93 ± 2.27^bdac^
	Treat15	4.40 ± 3.25^bdc^	5.17 ± 4.55^ba^	4.83 ± 3.81^bac^	3.10 ± 0.62^dc^	3.06 ± 0.36^d^	3.67 ± 1.88^bdc^
UrMg^++^ mg dL^−1^	Treat10	2.91 ± 0.10^a^	2.66 ± 0.42^bac^	2.60 ± 0.46^a^	2.11 ± 0.31^bc^	2.07 ± 0.20^c^	2.90 ± 0.10^bac^
	Treat15	2.71 ± 0.39^ba^	2.76 ± 0.22^ba^	2.41 ± 0.45^bac^	2.02 ± 0.15^c^	1.89 ± 0.05^c^	2.74 ± 0.33^bac^
UrCl^−^ mmol L^−1^	Treat10	136.51 ± 67.53^b^	98.84 ± 61.29^b^	73.70 ± 41.18^b^	102.03 ± 50.28^b^	107.60 ± 48.48^b^	333.51 ± 212.52^a^
	Treat15	119.35 ± 65.92^b^	90.44 ± 35.7^b^	93.35 ± 51.94^b^	82.79 ± 47.51^b^	91.27 ± 10.33^b^	245.05 ± 133.45^a^
UrNa^+^ mmol L^−1^	Treat10	1.00 ± 0.00^a^	2.80 ± 3.49^a^	2.25 ± 1.50^a^	23.00 ± 16.42^a^	33.50 ± 15.24^a^	2.50 ± 2.12^a^
	Treat15	2.50 ± 1.29^a^	2.00 ± 1.22^a^	1.80 ± 0.84^a^	20.83 ± 5.17^a^	38.83 ± 4.31^a^	6.60 ± 4.72^a^
UrK^+^ mmol L^−1^	Treat10	6.35 ± 0.26^ba^	5.28 ± 0.78^bc^	5.05 ± 1.14^bc^	4.68 ± 1.52^c^	5.53 ± 0.73^bac^	7.00 ± 0.00^a^
	Treat15	5.38 ± 0.96^bac^	4.72 ± 0.58^c^	5.56 ± 1.51^bac^	4.58 ± 1.13^c^	4.67 ± 1.22^c^	4.70 ± 0.19^c^

Values represented by different letters indicate statistical difference (*p* < 0.05).

Although there was an increase in urinary sodium excretion during treatment and urinary calcium excretion after treatment, these changes were not statistically significant (*p* > 0.05). Potassium excretion differed significantly between treatments (*p* = 0.0157), with higher values in Treat10 at T-24 and T24. Phosphorus and chloride excretion varied significantly over time (*p* = 0.0233 and *p* < 0.0001, respectively), with Cl^−^ peaking at T24, for both treatments. Magnesium (Mg^++^) also showed statistical (*p* = 0.0442) variations over time. Urea concentration in urine significantly changed over time (*p* = 0.0029), with a notable decrease during the fluid therapy period. Changes in urinary creatinine were not statistically significant (Table 4).

## 4 Discussion

The six horses successfully underwent enteral fluid therapy via the intracecal route in a continuous flow. One of the advantages of continuous flow administration is that it allows a steady and gradual infusion of electrolyte solutions, thereby avoiding distension of the digestive system and preventing the onset of discomfort or pain in the patient. No adverse effects were observed during the period that the cannulas remained in the animal’s cecum. Enteral electrolyte solutions were administered immediately after the surgical procedure (placement of the cannulas in the cecum). Both doses, 10 mL kg^−1^ h^−1^ (Treat10) and 15 mL kg^−1^ h^−1^ (Treat15), were well tolerated and did not cause any discomfort or pain in the animals throughout the fluid therapy phase (T0h to T12h), as evidenced by normal heart and respiratory rates and intestinal motility values (Table 1). No surgery-related difficulties or complications were recorded in any of the horses during the experimental phase.

Packed cell volume (PCV) and total serum protein (TSP) values remained unchanged after 24 h of fasting (T0h), indicating that this period was insufficient to cause significant increases in these parameters. During the fluid therapy phase (T4h to T12h), in animals from both groups, PCV and TSP values did not show a significant difference (Table 1). The absence of a decrease in the values of these two variables during the fluid therapy phase demonstrated that the administration time of the electrolyte solution was possibly insufficient, confirming that hydration is a continuous, gradual, and lengthy process. Similarly, Souza et al. (5) did not report changes in PCV and TSP after 24 h of fasting in adult horses and during 12 h of intracecal fluid therapy, corroborating the results of the present study.

No increases in abdominal circumference values were recorded in animals that received Treat10, while those that received Treat15 showed a slight but significant increase at T12h. This mild increase was possibly due to the volume of electrolyte solution infused into the animals. However, this slight distension was devoid of clinical significance, as the animals showed no signs of discomfort (Table 1).

The maintaining serum osmolarity within reference values (6) was an expected result, because there are adequate and effective hemodynamic compensation mechanisms. One of these mechanisms is performed by the kidneys. The reduction in urinary specific gravity may be one of the mechanisms of compensation. However, no significant decrease in urine specific gravity was observed during the fluid therapy period, although its values decreased (Table 4). Similar results to those of the present study were obtained by Souza et al. (5) when rehydrating adult horses via intracecal injection.

A decrease in urine-specific gravity is a direct indicator of hydration status, hemodilution, and urine dilution and has been reported in a variety of studies in adult horses and foals submitted to enteral fluid therapy (7–10). The lack of statistical significance reported in the present study may be attributed to individual variability and the consequent increase in standard deviation, as well as the small number of animals used. It should be emphasized that the visual assessment of urine dilution is limited and subjective.

Serum creatinine remained slightly below the reference range, potentially indicating suboptimal dietary protein intake (11). Urea increased after fasting and decreased with fluid therapy, with values exceeding the reference range only at T0, likely due to dehydration and subsequent hemodilution during fluid therapy (11). These findings are consistent with those reported in horses receiving enteral fluid therapy through the nasogastric route (7–9). The horses’ hydration status was maintained until T24, which is 12 h after the end of treatments. The final values are still lower than the initial ones, reaffirming the hydration potential of intracecal fluid therapy.

Urinary excretion of urea and creatinine decreased over time during fluid therapy, consistent with previous reports in horses receiving enteral fluid therapy (9). Although only changes in urea were statistically significant, both variables indicate urine dilution (10) caused by the administration of the enteral electrolyte solution (EES). Similar results for serum urea concentration and urinary excretion have been described in dehydrated horses and in horses subjected to enteral fluid therapy (7).

Studies on nasogastric enteral fluid therapy have suggested that this route is effective for restoring and maintaining plasma glucose levels in horses (7, 12, 13). In the present study, glucose concentrations remained unchanged in animals from both treatments. Plasma glucose levels did not decrease because the fasting period was relatively short, while the absence of an increase in glycemia was likely due to the small amount of dextrose in the electrolyte solution, combined with the short fluid therapy duration. The only exception was recorded at T24h (Table 2), when a slight increase in glucose levels was observed, likely reflecting the return of food access to the animals. Despite this minor increase, glucose levels remained within the normal physiological range (14). Weaned foals subjected to 12 h of fasting showed lower glucose values; however, after receiving nasogastric enteral fluid therapy (EFT) for 12 h, they recovered their glycemic levels efficiently (15).

Higher lactate values observed in Treat15 compared to Treat10 likely represent a pre-existing difference rather than a treatment effect, as values were elevated from baseline (T-24), before the animals were submitted to treatments, and lasted without variations until T24. Lactate levels remained within reference values (15), indicating that dehydration was not severe enough to induce anaerobic metabolism (7). Similar results have been described in horses and foals undergoing enteral fluid therapy with different compositions (15, 16).

Serum Na^+^ levels showed no significant variation throughout the experiment and remained within the reference range (6). Unaltered serum sodium concentrations were also reported in horses subjected to a 36-h dehydration protocol (7); these animals subsequently received three fluid therapy treatments-two with enteral electrolyte solutions (EES) and one with lactated Ringer’s solution. Although not statistically significant, a marked increase in urinary Na^+^ excretion was observed during fluid therapy. Foals undergoing 12 h of EFT also showed increased urinary sodium excretion (10). In adult horses, sodium excretion increased both as a result of dehydration and following fluid therapy with lactated Ringer’s solution (7). Conversely, sodium excretion in horses treated with three different EES formulations containing various carbohydrate precursors did not show significant changes (9). Sodium is efficiently absorbed through the cecal and colonic mucosa. Therefore, the Na^+^ contained in the EES may have been fully absorbed by the animals. To maintain homeostasis, the kidneys regulate sodium concentrations primarily via urinary excretion (17). Horses are highly efficient at reabsorbing Na^+^ (18), suggesting that the observed increase in urinary excretion was likely a response to the elevated sodium concentration in the administered solution. This finding indicates that the sodium content of the formulation could be optimized in future studies.

Serum Cl^−^ levels did not show statistically significant variations and remained within reference ranges throughout the study (19). In contrast, horses subjected to a dehydration protocol using furosemide exhibited significantly decreased serum Cl^−^ concentrations. The authors attributed this finding to the pharmacological effect of furosemide. Furthermore, they reported that both enteral fluid therapy (EFT) and intravenous fluid therapy were effective in restoring chloride levels to normal (7). Chloride is absorbed in multiple regions of the large intestine, typically in conjunction with sodium. Additionally, the kidneys reabsorb approximately 90% of the Cl^−^ filtered by the glomeruli (6, 19). In the present study, urinary Cl^−^ excretion remained stable across most experimental time points but showed a marked increase during the final evaluation, 12 h after the end of intracecal fluid therapy. Despite this substantial urinary excretion at T24, a slight rise in serum Cl^−^ concentration was also observed at the same time. This could be attributed to the high chloride concentration in the administered enteral electrolyte solution, as previously noted in other studies (15, 20). The administration of certain fluids has been shown to increase both serum levels and renal excretion of Cl^−^ (17, 21). For example, horses that received 8 h of lactated Ringer’s solution exhibited elevated urinary chloride excretion as a compensatory response to the solution’s high chloride content (6). Another possible explanation is the increased intake of mineral salts following the prolonged fasting period. Since feeding was resumed immediately after T12 and the elevated chloride values were recorded at T24, it is plausible that the increased serum and urinary Cl^−^ levels were related to dietary intake rather than the fluid therapy itself.

Serum iCa^++^ levels remained within the reference range (22) and showed no significant variation over time, indicating that the iCa^++^ concentration in the EES was adequate. Enteral electrolyte solutions have been reported to be more effective than Lactated Ringer’s solution in restoring serum iCa^++^ concentrations in horses (7). Horses exhibit high efficiency in intestinal iCa^++^ absorption, with approximately 75% of dietary calcium absorbed, while any excess is excreted in the urine (22, 23). During fluid therapy, urinary iCa^++^ excretion remained stable across all animals. However, at T-24 and T24, increased standard deviations were observed. This variation in urinary calcium excretion, although not statistically significant, may be related to the horses’ feeding behavior (22, 23), as the administration of the solution was controlled, while access to feed was ad libitum.

Serum Mg^++^ concentrations remained consistently below the reference ranges (6) and showed a slight, though not statistically significant, decrease during fluid therapy. Serum magnesium levels are typically influenced by dietary intake, with approximately 60% of absorption occurring in the small intestine and only about 5% in the large intestine (24). Given this, it is reasonable to assume that Mg^++^ supplementation via intracecal fluid therapy may be less effective than via the nasogastric route. Previous studies have emphasized the need to increase Mg^++^ concentrations in enteral electrolyte solutions (15, 16). Furthermore, both enteral electrolyte solution and lactated Ringer’s solution have been reported to provide insufficient magnesium to support fasted horses (6). The urinary excretion curve for Mg^++^ mirrored its serum concentration pattern, suggesting the maintenance of tightly regulated homeostasis. Another hypothesis is that the reference ranges cited in the literature may not accurately reflect the population used in this study, especially since no clinical signs of hypomagnesemia were observed. Considering that approximately 80% of Mg^++^ is reabsorbed by the kidneys (24), a true deficiency would likely have resulted in a progressive decline in renal excretion.

The serum concentration of K^+^ showed a marked decrease during fluid therapy. Anorectic horses typically present with hypokalemia (23). The K^+^ values observed in this study suggest that intracecal fluid therapy was insufficient to maintain adequate serum levels, likely due to the 36-h fasting period to which the horses were subjected. Studies on nasogastric enteral fluid therapy have recommended higher K^+^ concentrations for anorectic animals (16). Horses subjected to similar fasting protocols also showed reduced serum K^+^ concentrations (6). However, in contrast to the present study, those cases successfully restored K^+^ levels using nasogastric or intravenous fluid therapy (7). This difference may be attributed to the fact that potassium absorption occurs predominantly in the small intestine rather than the large intestine (25). Despite the decline, K^+^ levels in this study remained within the reference range at all times (6). Regarding urinary excretion, significant differences were observed between treatments, particularly at T-24 and T24. In horses subjected to prolonged fasting, renal reabsorption of K^+^ tends to increase (17), which may explain the reduced urinary excretion at T0 and the subsequent rise following the reintroduction of food. Additionally, lower urinary K^+^ concentrations may result from urine dilution caused by fluid therapy (9).

Serum Pi concentrations in this study remained consistently below the reference range for horses (6). However, they were stable throughout the experimental period, except at T24h, when values decreased in animals from both treatments (Table 1). The enteral electrolyte solution used in the treatments did not contain a phosphorus source. Fluctuations in this element are likely associated with its interactions with other electrolytes and may also reflect the effects of hemoconcentration or hemodilution (7, 16). Conversely, urinary phosphorus (UrP) values remained stable throughout the experimental phase, except at T0h, when an increase was observed. This rise was likely due to the higher urinary concentration resulting from the fasting period imposed on the animals.

Animals with low Mg^2+^ levels often exhibit reduced Pi concentrations due to the close metabolic relationship between these electrolytes (26), and the findings of this study support this association. Serum Mg concentrations remained unchanged but below the reference range (14) in animals from both treatments. Urinary magnesium (UrMg) values showed slight variations throughout the experimental phase in both treatment groups (Table 4).

Venous blood pH and pCO₂ remained within reference limits and did not show statistically significant differences (5). However, HCO₃^−^ and base excess concentrations decreased in both treatments at T24h. Because twelve hours had elapsed since the end of fluid therapy, the observed decrease in these two variables was likely related to the resumption of feeding, particularly due to the inclusion of concentrate in the diet provided to the animals. This reduction can be attributed to the mobilization of the buffering system to maintain pH homeostasis (21). It is also important to note that the administration of an enteral electrolyte solution containing a high amount of carbohydrates can promote fermentation by the intestinal microbiota and the subsequent production of organic acids, thereby increasing the physiological demand for bicarbonate (HCO₃^−^) as a buffer (20).

Urinary pH changes are expected as the kidneys work to maintain acid–base balance. The kidneys achieve this by regulating the excretion and absorption of various components (17). One of the major mechanisms is the control of H^+^ and Cl^−^ excretion and reabsorption (20). The pattern observed in this study, where urinary pH decreases, Cl^−^ excretion increases, and blood HCO₃^−^ levels decrease (all at T24), resembles that of renal tubular acidosis (20). However, as no findings indicated renal disease in the horses, it is likely that this was an attempt to regulate acid–base balance. For instance, mild aciduria may occur as a result of the H^+^ excretion regulatory mechanism (7). Previous studies using enteral electrolyte solutions with different carbohydrate sources also did not report significant alterations in urinary pH in adult horses (8), although mild aciduria was observed.

Because most of the variables used to calculate the anion gap and the strong ion difference did not exhibit significant statistical variation, it was expected that these parameters would behave similarly. However, the strong ion difference showed a slight decrease at T24h, which coincided with the peak in Cl^−^ concentration. Reductions in SID are commonly associated with hyperchloremia and its contribution to systemic acidification (21).

The main limitations of this study were the small number of animals used, the short fasting period imposed, and the limited number of variables measured to accurately assess the effects of dehydration induced by food and water deprivation, as well as the subsequent rehydration process. Furthermore, extending the rehydration period would provide a better understanding of the physiological effects of intracecal fluid therapy over time.

## 5 Conclusion

Intracecal administration of electrolyte solutions in a continuous flow at rates of 10 mL kg^−1^ h^−1^ (Treat10) and 15 mL kg^−1^ h^−1^ (Treat15) for 12 h was safe and effective for the animals, although a mild increase in abdominal circumference was seen in the animals that received the Treat15. Furthermore, the enteral electrolyte solution used in this study did not cause significant alterations in electrolyte or acid–base balance, suggesting that its composition is appropriate. These results demonstrate that intracecal fluid therapy can be considered a viable, effective, and safe option for use in horses, nevertheless further studies are needed.

## Data Availability

The original contributions presented in the study are included in the article/supplementary material, further inquiries can be directed to the corresponding authors.
